# Omitting face-mask ventilation before tracheal intubation in low-risk adults: a randomized non-inferiority trial

**DOI:** 10.1186/s12871-026-04276-y

**Published:** 2026-09-19

**Authors:** Hyoung Woo Chang, Namo Kim, Darhae Eum, Hae Dong Kim, Joung Goo Cho, Hyun Joo Kim

**Affiliations:** 1 Department of Thoracic and Cardiovascular Surgery, Seoul National University Bundang Hospital, Seoul National University College of Medicine, Gyeonggi-do, Seongnam-si, 13620 Republic of Korea; 2 Department of Anesthesiology and Pain Medicine, Anesthesia and Pain Research Institute, Severance Hospital, Yonsei University College of Medicine, Seoul, 03722 Republic of Korea; 3 Department of Anesthesiology and Pain Medicine, National Health Insurance Service Ilsan Hospital, Goyang-si, Gyeonggi-do 10444 Republic of Korea; 4 Department of Anesthesiology and Pain Medicine, Anesthesia and Pain Research Institute, Gangnam Severance Hospital, Yonsei University College of Medicine, Seoul, 06273 Republic of Korea

**Keywords:** Face-mask ventilation, Tracheal intubation, Gastric insufflation, Rocuronium, Preoxygenation, Gastric ultrasonography

## Abstract

**Background:**

Face-mask ventilation (FMV) is routinely performed between the induction of general anesthesia and tracheal intubation, but it can cause gastric insufflation, and its necessity in low-risk patients is uncertain. We examined whether omitting FMV before intubation is non-inferior to routine FMV for oxygen desaturation, and whether the two approaches differ in gastric insufflation.

**Methods:**

In this single-center, randomized, group-sequential non-inferiority trial, low-risk adults undergoing elective thyroid surgery were assigned to tracheal intubation with FMV (rocuronium 0.6 mg/kg) or without FMV (rocuronium 1.2 mg/kg). The primary outcome was the incidence of desaturation (peripheral oxygen saturation [SpO₂] ≤ 95%) from induction to the first intubation attempt, with a non-inferiority margin of 2% (absolute risk difference). The pre-specified secondary outcome was the gastric antral cross-sectional area (CSA) after intubation, measured by ultrasonography. The trial began as a fixed-sample design; a single interim analysis at 50% of enrollment (O’Brien–Fleming spending function) was added by protocol amendment after enrollment had begun. Gastric insufflation, defined by a qualitative ultrasonographic sign and by an antral CSA increase greater than 30%, was analyzed post hoc.

**Results:**

The trial was stopped at the interim analysis with 288 participants (144 per group), a decision made in the absence of any events rather than by a stopping rule. No patient in either group reached the desaturation threshold (0% vs. 0%; risk difference 0%, 95% confidence interval − 2.6% to 2.6%). The gastric antral CSA after intubation did not differ between groups (median 3.7 cm² in both groups; *P* = 0.99). In post hoc analyses, gastric insufflation occurred only in the FMV group, both by the qualitative sign (8/144 [5.6%] vs. 0/144; *P* = 0.007) and by the antral CSA threshold (9/144 [6.2%] vs. 0/144; *P* = 0.003).

**Conclusions:**

In low-risk adults undergoing elective thyroid surgery, no patient reached the desaturation threshold whether or not FMV was performed before intubation. Because the trial was stopped at the interim analysis with zero events, the upper limit of the confidence interval for the risk difference exceeded the pre-specified 2% margin, and formal non-inferiority was not established. In exploratory post hoc analyses, gastric insufflation occurred only in the FMV group. These hypothesis-generating findings warrant confirmation in adequately powered trials before routine FMV is omitted in low-risk patients.

**Trial registration:**

ClinicalTrials.gov, NCT02400931. Registered 23 March 2015.

## Background

After the induction of general anesthesia and before tracheal intubation, face-mask ventilation (FMV) is performed routinely, often as a matter of habit. It serves two purposes: to maintain oxygenation during the apneic interval until intubating conditions are achieved, and to confirm that the lungs can be ventilated before or around the administration of a neuromuscular blocking agent. However, FMV increases the incidence of gastric insufflation [1], which raises intragastric pressure and may predispose to regurgitation and impair ventilation. Whether routine FMV before intubation is necessary in patients at low risk for airway difficulty has rarely been examined.

In low-risk patients, however, neither purpose may be necessary. The practice of demonstrating adequate FMV before giving a neuromuscular blocker has been directly challenged [2] and remains common despite little evidence that it improves safety [3]. Neuromuscular blockade does not impair, and may improve, FMV, including in patients who are initially difficult to ventilate [4, 5]; difficult-airway guidelines accordingly recommend establishing neuromuscular blockade when ventilation is failing rather than withholding it [6, 7]. It follows that confirming ventilation before neuromuscular blockade is unnecessary, removing one of the two reasons for performing FMV.

The oxygenation rationale is also weaker than it appears. Thorough preoxygenation builds oxygen stores that delay arterial desaturation during apnea [8, 9], and apneic oxygenation can extend the safe apnea period well beyond the time needed to intubate [10, 11]. In parallel, a high dose of rocuronium produces intubating conditions within approximately 1 min, while sugammadex permits rapid reversal should rescue be required [12]. Together, these advances make it plausible that, after thorough preoxygenation, a low-risk patient could be intubated safely without any intervening FMV.

Whether FMV can be omitted altogether, however, has rarely been examined. Prior randomized trials compared the timing of FMV relative to neuromuscular blockade rather than its omission [4, 5]. We therefore conducted a randomized non-inferiority trial in low-risk adults to determine whether omitting FMV before tracheal intubation is non-inferior to routine FMV for oxygen desaturation, with the gastric antral cross-sectional area, assessed by ultrasonography, as the pre-specified secondary outcome.

## Methods

### Study design and setting

This was a randomized, parallel-group, group-sequential non-inferiority trial conducted at Severance Hospital, Yonsei University College of Medicine, Seoul, Republic of Korea. The trial was performed in accordance with the Declaration of Helsinki and is reported in accordance with the CONSORT statement and its extension for non-inferiority trials. The protocol was approved by the Institutional Review Board of Severance Hospital, Yonsei University Health System (approval no. 4-2015-0036; approved March 18, 2015) and was registered at ClinicalTrials.gov before patient enrollment (NCT02400931; registered March 23, 2015). The trial began as a fixed-sample non-inferiority design. A single interim analysis at 50% of the target enrollment, with stopping guided by an O’Brien–Fleming spending function, was added by an Institutional Review Board–approved protocol amendment submitted in August 2015—during enrollment but before any interim outcome data were examined. Approximately 190 of the eventual 288 participants had been enrolled at that point, and the interim analysis itself was performed only after enrollment was complete. Written informed consent was obtained from every participant in a private setting.

A permuted-block randomization list (block size of four; allocation ratio 1:1) was generated with an online randomizer (numbers 1 and 2) by a third party not otherwise involved in the trial. The sequence was concealed in sealed, sequentially numbered envelopes that were opened by the attending anesthesiologist only after the patient had entered the operating room, at which point the group assignment was revealed. Because the two strategies are overtly different, the attending anesthesiologist could not be blinded to group allocation; however, the investigator who measured the antral dimensions and assessed the ultrasonographic gas sign on the stored images was blinded to group allocation.

### Patients

Adults aged 20 years or older scheduled for elective thyroid surgery under general anesthesia expected to last at least 90 min were eligible.

Patients were excluded if difficult mask ventilation or difficult tracheal intubation was anticipated: a history of difficult intubation, modified Mallampati class 3 or 4, an inter-incisor distance less than 3 cm, limited neck extension, inability to protrude the lower incisors beyond the upper incisors, or a thyromental distance less than 6 cm. Patients in whom a goiter was large enough to distort the airway—for example, through tracheal deviation or compression—were excluded under these criteria. Patients were also excluded for an American Society of Anesthesiologists (ASA) physical status of 3 or higher due to valvular heart disease, congestive heart failure, chronic obstructive pulmonary disease, or pneumonia; a peripheral oxygen saturation (SpO₂) of 92% or less before preoxygenation; an SpO₂ below 99% after preoxygenation; a body mass index greater than 30 kg/m²; diagnosed obstructive sleep apnea; an oral or airway tumor; and pregnancy.

### Intervention

In the operating room, patients were placed supine with a pillow under the head, and a bispectral index sensor was applied to monitor the depth of anesthesia. Preoxygenation was performed for 3 min via a tight-fitting anesthesia facemask with 100% oxygen at a fresh gas flow of 6 L/min. Anesthesia was induced with propofol (1.5–2 mg/kg) and either a remifentanil infusion (0.1–0.15 µg/kg/min) or fentanyl (1–1.5 µg/kg).

In the FMV group, after loss of consciousness, rocuronium 0.6 mg/kg was administered, and FMV was performed from immediately after rocuronium until there was no response to train-of-four (TOF) stimulation of the ulnar nerve. The anesthesiologist held the mask with both hands and applied a jaw thrust. FMV was delivered with a mechanical ventilator in pressure-controlled mode set to a tidal volume of 8 mL/kg, a fresh gas flow of 2 L/min, and a respiratory rate of 15 breaths/min, without added positive end-expiratory pressure (PEEP). Tracheal intubation was performed when there was no response to TOF stimulation.

In the no-FMV group, rocuronium 1.2 mg/kg was administered after propofol, FMV was not performed, and tracheal intubation was performed when there was no response to TOF stimulation. No supplemental oxygen was administered during the apneic interval. If SpO₂ decreased to 95% or less before intubation, FMV was started immediately with the settings described above.

In both groups, tracheal intubation was performed by direct laryngoscopy. If intubation failed, FMV was provided in all patients regardless of group and intubation was reattempted. If SpO₂ did not recover to 90% or more despite FMV, management followed the American Society of Anesthesiologists Practice Guidelines for Management of the Difficult Airway [13], and the participant was withdrawn with no further data collected. Sugammadex was immediately available in the operating room and could be administered without delay, at the standard dose for reversal of rocuronium, if rapid re-establishment of spontaneous ventilation was required.

### Outcome measures

#### Primary outcome

The primary outcome was the incidence of desaturation, defined as an SpO₂ of 95% or less at any point from the induction of anesthesia to the first attempt at tracheal intubation.

#### Secondary outcome

The secondary outcome was the gastric antral cross-sectional area (CSA) after tracheal intubation. With the patient supine, a transverse view of the gastric antrum was obtained, the craniocaudal (CC) and anteroposterior (AP) diameters of a single section were measured, and the antral CSA was calculated as (π × CC × AP) / 4 [14]. The antral CSA was recorded three times before induction and three times after intubation, and the mean of the three measurements was used.

### Data collection

The following data were also recorded: age, sex, height, and weight; ASA physical status, modified Mallampati class, inter-incisor distance, and thyromental distance; the time from rocuronium to no response to TOF stimulation; the time from rocuronium to the first detection of end-tidal carbon dioxide after intubation; and, in the FMV group, the peak inspiratory pressure over five consecutive breaths beginning with the fifth breath. The lowest SpO₂ before the first intubation attempt and the lowest SpO₂ within 2 min after intubation were recorded; the SpO₂ before and after the 3-min preoxygenation were also recorded and were used to confirm eligibility. After emergence, patients were asked whether they recalled the induction. The anesthesiologist who performed the induction rated, on a 0–10 visual analog scale (VAS), the fear of desaturation during induction, the satisfaction with omitting FMV (no-FMV group), and the arm fatigue caused by FMV (FMV group). These operator-reported ratings were exploratory, unvalidated, and assessed without blinding, and are interpreted with corresponding caution.

### Statistical analysis

The primary comparison was a non-inferiority test of the risk difference in the incidence of desaturation (no-FMV minus FMV). The non-inferiority margin was pre-specified at a 2% absolute increase in the incidence of transient desaturation (SpO₂ ≤ 95%, a mild and readily reversible threshold), the largest difference judged clinically acceptable in exchange for omitting FMV. The anticipated incidence of desaturation in the FMV group was assumed to be 0.5%. Direct data on transient desaturation during this specific interval were unavailable, so this estimate was derived from the reported incidence of mask-ventilation failure, which is defined in part by inability to maintain oxygenation: grade 3 (“difficult”) mask ventilation is defined as ventilation “inadequate to maintain oxygenation, unstable, or requiring two providers” [15]. In large cohorts that included patients with difficult-airway predictors, impossible mask ventilation occurred in 0.15% of approximately 53,000 patients [16] and difficult or inadequate mask ventilation in 1.56% of 22,660 patients [15]; because such predictors were exclusion criteria in the present trial, a lower value (0.5%) was chosen as a conservative estimate.

The trial used a group-sequential design with a single interim analysis at 50% of enrollment (added by the August 2015 amendment described above), using an O’Brien–Fleming spending function. The maximum sample size was computed by simulation in PASS version 12 (NCSS, LLC, Kaysville, UT, USA; “Group-Sequential Non-Inferiority Tests for Two Proportions using Differences [Simulation],” 10,000 simulations) at a one-sided significance level of 0.05, assuming the 0.5% incidence, the 2% margin, and a target power of 80%; this yielded 287 participants per group (574 in total), with the interim analysis planned at 144 participants per group (288 in total). For the primary analysis, non-inferiority was assessed with the two-sided 95% confidence interval (equivalent to a one-sided level of 0.025), as is conventional for non-inferiority trials. The level used for the analysis therefore differed from the one-sided 0.05 used to plan the maximum sample size, and the two are not interchangeable: at 287 participants per group the design provided approximately 78% power at a one-sided 0.05 but only about 61% at a one-sided 0.025, and roughly 388 participants per group would have been required for 80% power at the level actually applied. The trial was thus underpowered relative to its own primary analysis even at full enrollment; because no events occurred this did not affect the present result, as the full-sample upper confidence limit would still approach 1%. The interim analysis was performed by an independent statistical team, and trial conduct and participant safety were overseen by an independent safety monitor.

All randomized participants were analyzed as assigned (intention-to-treat); because there were no losses to follow-up or protocol deviations, the per-protocol population was identical. Continuous variables were assessed for normality with the Shapiro–Wilk test and are presented as mean ± standard deviation or median [interquartile range]; categorical variables are presented as number (%). Between-group comparisons of continuous variables used the independent t-test or the Mann–Whitney U test according to distribution, and comparisons of categorical variables used the chi-square test or Fisher’s exact test. For the primary outcome, non-inferiority was to be concluded if the upper limit of the two-sided 95% confidence interval (CI) of the risk difference (no-FMV minus FMV) was below the 2% margin; because no desaturation events occurred in either group, the risk difference and its 95% CI were estimated with the Miettinen–Nurminen score method, for which a Wald interval is undefined. With zero events, the interval is method-dependent, and we therefore also report the Newcombe and Agresti–Caffo intervals. The pre-specified secondary outcome was compared with the Mann–Whitney U test.

Two binary measures of gastric insufflation were analyzed post hoc and were not pre-specified: a qualitative ultrasonographic sign (a new comet-tail artifact and/or acoustic shadow indicating intragastric gas [1]) and an objective threshold of an increase of more than 30% in antral CSA between the pre-induction and post-intubation measurements, used to corroborate the qualitative sign with a reproducible quantitative criterion. These binary measures were compared with Fisher’s exact test, with the risk difference and its 95% CI estimated by the Miettinen–Nurminen score method, and are reported as exploratory and hypothesis-generating. In additional post hoc analyses that were not pre-specified, we assessed the concordance between the two binary measures, repeated the threshold count at 20% and 40% as a sensitivity analysis, and compared the within-patient change in antral CSA between groups with the Mann–Whitney U test, summarizing the between-group difference with the Hodges–Lehmann median difference and its 95% confidence interval. A two-sided P value below 0.05 was considered statistically significant, and no adjustment for multiplicity was applied. Analyses were performed with Python 3.9 (pandas 2.3, SciPy 1.13, and statsmodels 0.14); the sample size was computed with PASS version 12.

## Results

### Patient flow and characteristics

**Fig. 1:**
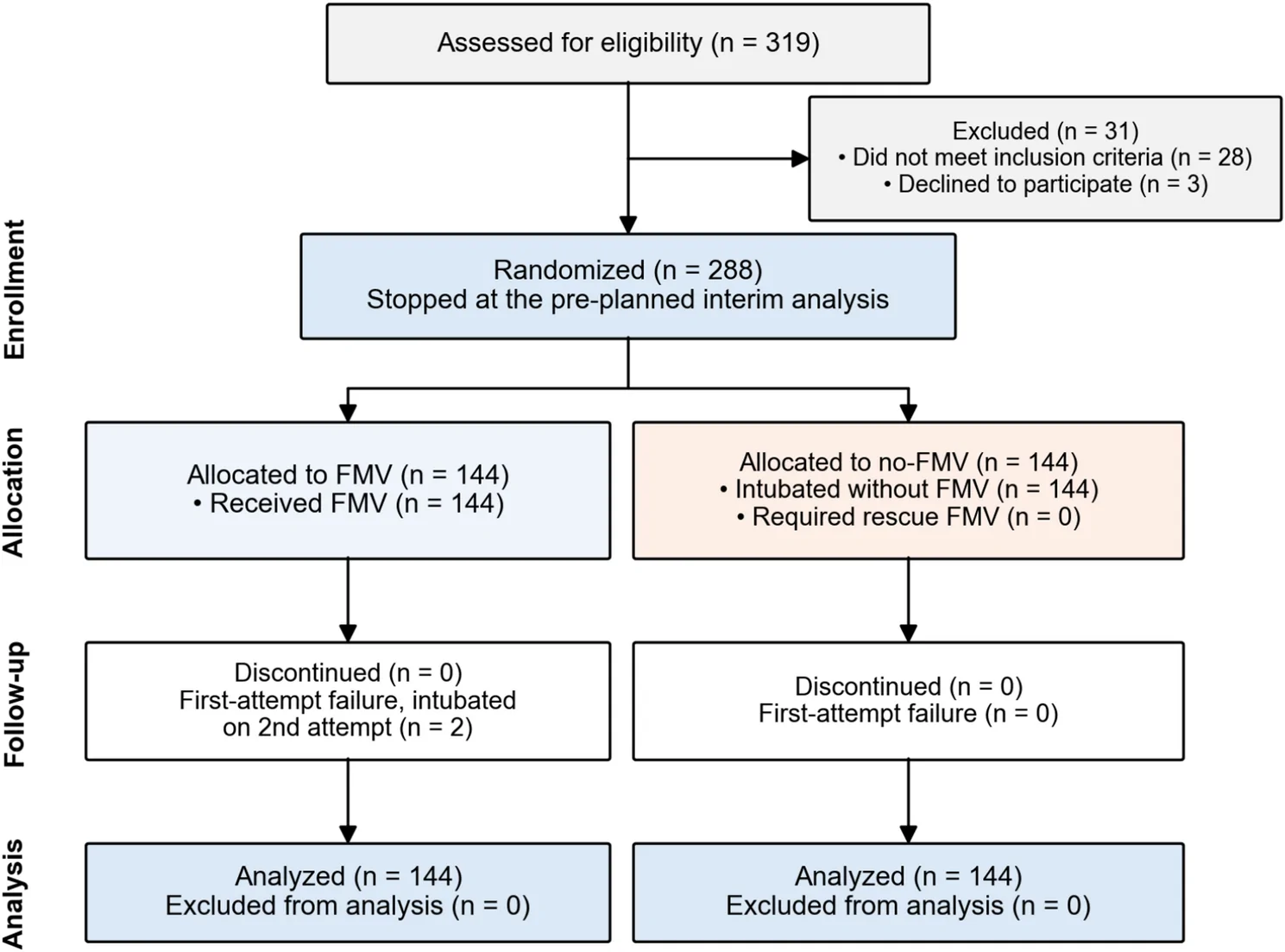
CONSORT flow diagram. FMV, face-mask ventilation

Between April 28, 2015 and November 23, 2015, 319 patients were assessed for eligibility; 31 were excluded and 288 were randomized to the FMV group (*n* = 144) or the no-FMV group (*n* = 144). The trial was stopped at the interim analysis, when no desaturation event had occurred in either group; because there were zero events, no stopping boundary was crossed, and enrollment was halted as a judgement made in the absence of any events rather than as an action mandated by the pre-specified rule. All 288 randomized participants were analyzed with no exclusions (Fig. 1). The eligibility criterion of an SpO₂ of at least 99% after preoxygenation could be verified only after the patient entered the operating room and allocation had been revealed; it was applied identically in both groups, and every randomized participant met it (post-preoxygenation SpO₂ 100% in all 288), so no participant was excluded after randomization on this basis (Fig. 1). Baseline characteristics were similar between the groups, and most participants were women, consistent with an elective thyroid-surgery population (Table 1). All participants were of ASA physical status I or II with a modified Mallampati class of 1 or 2, consistent with the low-risk eligibility criteria.

**Table 1:** Baseline characteristics. Data are presented as mean±standard deviation and frequency (%)

Characteristic	FMV (*n* = 144)	No-FMV (*n* = 144)	*P* value
Age, yr	41.7 ± 9.3	42.5 ± 9.9	0.489
Female sex	138 (95.8)	125 (86.8)	0.007
Height, cm	160.0 ± 6.6	160.9 ± 6.3	0.125
Weight, kg	56.9 ± 7.9	57.5 ± 7.2	0.333
Body mass index, kg/m²	22.2 ± 2.6	22.2 ± 2.4	0.942
Modified Mallampati II	48 (33.3)	43 (29.9)	0.526
Inter-incisor distance, cm	3.8 ± 0.5	3.8 ± 0.4	0.632
Thyromental distance, cm	6.6 ± 0.4	6.6 ± 0.4	0.251
ASA physical status II	6 (4.2)	9 (6.2)	0.426

*ASA* American Society of Anesthesiologists, *FMV* face-mask ventilation

### Primary outcome

No patient in either group had an SpO₂ of 95% or less between induction and the first intubation attempt, giving zero events in both groups (risk difference 0%; 95% CI, − 2.6% to 2.6%; Table 2). Because both groups had zero events, the upper limit of the 95% CI (2.6%) exceeded the pre-specified 2% margin, and non-inferiority was not formally established; the interval was also method-dependent (upper limit 2.6% by the Newcombe method and 1.9% by the Agresti–Caffo method). The lowest SpO₂, both before the first intubation attempt and within 2 min after intubation, had a median of 100% in both groups (interquartile range 100–100%); the lowest value observed in any participant was 98% (in the no-FMV group), and no participant in the no-FMV group required rescue FMV.

**Table 2:** Primary endpoint and pre-specified secondary endpoint. Data are presented as frequency (%) and medians (interquartile range)

Endpoint	FMV (*n* = 144)	No-FMV (*n* = 144)	Effect (95% CI)	*P* value
Desaturation (SpO₂ ≤ 95%), *n* (%)	0 (0.0)	0 (0.0)	RD 0.0% (− 2.6 to 2.6)	—
Antral CSA before induction, cm²	3.8 [3.0–4.4]	3.9 [3.1–4.3]	—	0.923
Antral CSA after intubation, cm²	3.7 [3.0–4.4]	3.7 [3.1–4.4]	—	0.989

*CSA* cross-sectional area, *CI* confidence interval, *FMV* face-mask ventilation, *RD* risk difference (no-FMV − FMV)

### Secondary outcome

The gastric antral CSA after intubation did not differ between the groups; antral CSA was also similar between the groups before induction (Table 2).

### Post hoc analyses

**Fig. 2:**
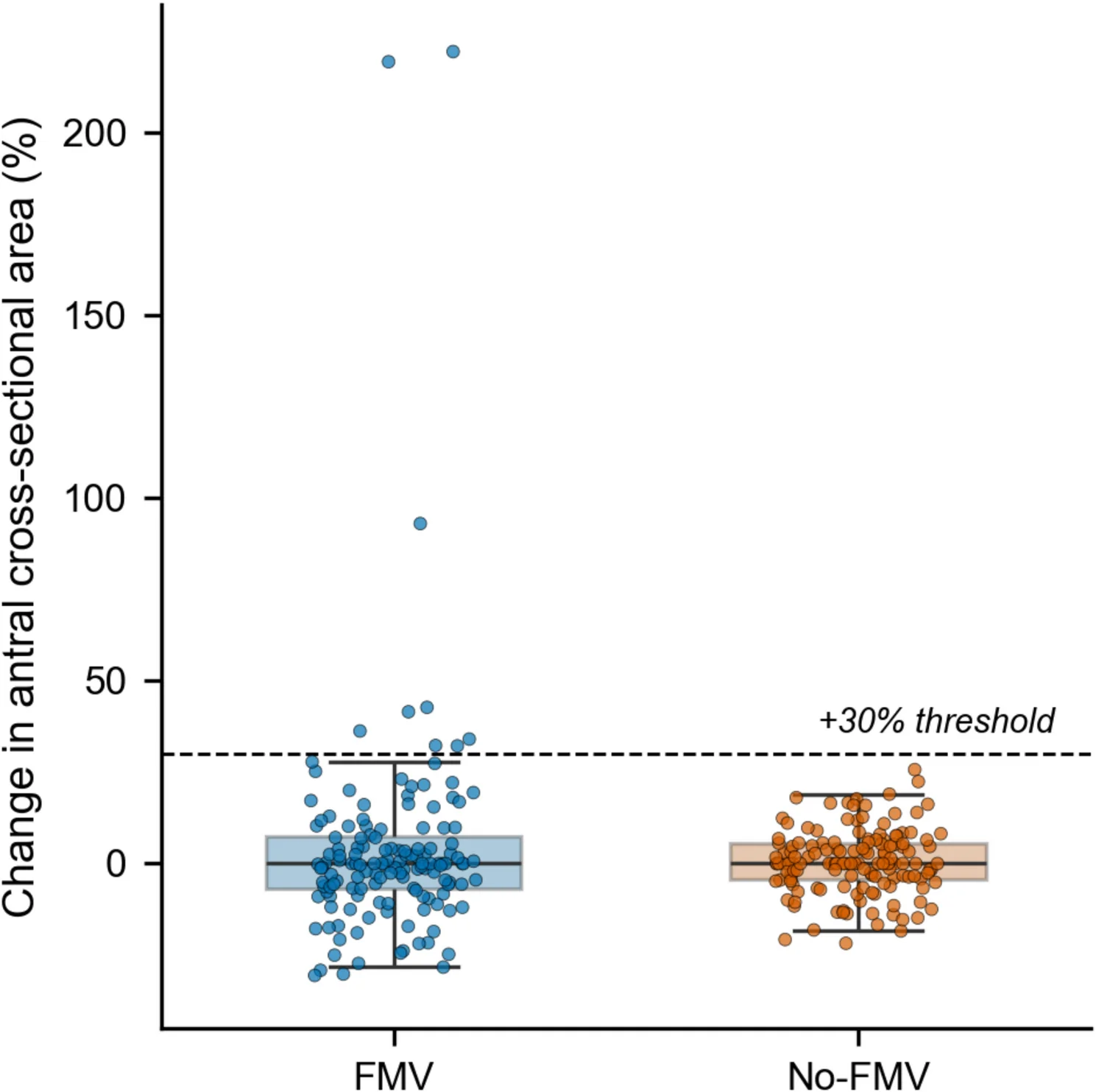
Change in gastric antral cross-sectional area after induction of anesthesia between the pre-induction and post-intubation measurements, by group. Each point is one participant; boxes show the median and interquartile range and whiskers the 1.5 × IQR range. The dashed line marks the post hoc +30% threshold used to define gastric insufflation. Increases above this threshold occurred only in the FMV group (9 of 144), whereas no participant in the no-FMV group exceeded the threshold. FMV, face-mask ventilation; IQR, interquartile range

In two post hoc, exploratory analyses that were not pre-specified, both the qualitative ultrasonographic sign and the objective threshold of an antral CSA increase greater than 30% occurred only in the FMV group and in no participant in the no-FMV group (Table 3; Fig. 2). The two measures were largely concordant: 7 of the 8 participants with the qualitative sign were among the 9 who exceeded the 30% threshold. Because the 30% cut-point was arbitrary, we repeated the count at alternative thresholds; an antral-CSA increase occurred in 17 versus 2 participants at 20% and in 5 versus 0 at 40% (FMV vs. no-FMV), so the direction of the finding did not depend on the chosen cut-point. In a further analysis that was not pre-specified, the between-group difference in the within-patient change in antral CSA, treated as a continuous outcome (Fig. 2), was not significant (median change 0% in both groups; Hodges–Lehmann median difference − 0.5%, 95% CI − 3.0% to 1.8%; *P* = 0.58); the binary signal thus reflected a right-tailed minority of patients rather than a shift in the overall distribution. These findings are hypothesis-generating.

**Table 3:** Gastric insufflation (post hoc, not pre-specified). Data are presented as frequency (%)

Measure	FMV (*n* = 144)	No-FMV (*n* = 144)	Risk difference (95% CI)	NNT (95% CI)	*P* value
Qualitative ultrasound sign, *n* (%)	8 (5.6)	0 (0.0)	5.6% (2.8 to 10.6)	18 (9 to 36)	0.007
Antral CSA increase > 30%, *n* (%)	9 (6.2)	0 (0.0)	6.2% (3.3 to 11.5)	16 (9 to 30)	0.003

*CSA* cross-sectional area, *FMV* face-mask ventilation, *NNT* number need-to-treat

### Procedural and operator-reported measure

The time from rocuronium to loss of TOF response and the time to the first end-tidal CO₂ were longer in the FMV group, consistent with the lower rocuronium dose used in that group (Table 4). First-attempt intubation success did not differ between the groups; the two first-attempt failures, both in the FMV group, were intubated on the second attempt (Table 4). Operator-rated fear of desaturation was low in both groups but slightly higher when FMV was omitted (Table 4); these operator-reported ratings were exploratory, unvalidated, and unblinded and are not interpreted further. Peak inspiratory pressure during FMV and operator-rated arm fatigue were recorded in the FMV group, and operators reported high satisfaction with omitting FMV in the no-FMV group (Table 4).

**Table 4:** Procedural and operator-reported measures. Data are median [IQR] or n (%)

Measure	FMV (*n* = 144)	No-FMV (*n* = 144)	*P* value
Procedural			
Time to loss of train-of-four, s	169 [130–194]	95 [73–114]	< 0.001
Time to first end-tidal CO₂, s	216 [182–252]	142 [120–165]	< 0.001
First-attempt intubation success, *n* (%)	142 (98.6)	144 (100.0)	0.498
Peak inspiratory pressure during FMV, cmH₂O	10 [10–10] (range 7–14)	—	—
Operator-reported (VAS, 0–10)			
Fear of desaturation	0 [0–0]	0 [0–1]	0.007
Satisfaction with omitting FMV	—	9 [7–9]	—
Arm fatigue from FMV	3 [2–4]	—	—

*VAS* visual analog scale, *FMV* face-mask ventilation

For fear of desaturation, both medians were 0; the significant *P* value reflects a difference in the distributions rather than in the medians

### Adverse events

No participant was withdrawn for failure to maintain oxygenation, no desaturation event occurred, and no participant reported recall of the induction.

## Discussion

In this randomized trial of low-risk adults undergoing elective thyroid surgery, omitting FMV before tracheal intubation after a high dose of rocuronium was not accompanied by any episode of oxygen desaturation: no patient in either group reached the desaturation threshold, and the lowest oxygen saturation showed a pronounced ceiling effect. Gastric insufflation, by contrast, was seen only when FMV was performed.

Clinically, these findings suggest that FMV can be omitted before intubation in low-risk adults without compromising oxygenation. We regard this as reassuring rather than confirmatory, however, because the trial closed at the interim analysis with no events in either group and non-inferiority was therefore not formally established.

That oxygenation was preserved without FMV is physiologically unsurprising. After adequate preoxygenation, the safe apneic period in healthy adults extends well beyond the brief interval needed to intubate after a high dose of rocuronium [8, 9]. In a randomized study of ASA physical status I–II adults that applied the same SpO₂ ≤ 95% threshold, oxygen saturation in the control group, which received no supplemental oxygen during apnea, did not fall to 95% until a mean of approximately 3.6 min [10]—longer than the time to intubation in our no-FMV group.

The gastric insufflation we observed is notable because it occurred despite a deliberately low airway pressure. The peak inspiratory pressure during pressure-controlled FMV was a median of 10 cmH₂O (interquartile range, 10–10; full range 7–14), below the 15–20 cmH₂O usually recommended to limit insufflation and below the resting barrier pressure of the lower esophageal sphincter. Gas nonetheless entered the stomach in about 6% of patients. This is roughly a third of the approximately one-fifth reported by Bouvet et al. at a comparable inspiratory pressure of 10 cmH₂O [1]; the lower incidence in our trial is most plausibly explained by the fact that their patients were not paralysed, together with differences in the ultrasonographic definition of insufflation. The patients with insufflation had inspiratory pressures within the same low range as the rest (median 10 cmH₂O, range 8–14), indicating that gas entered the stomach at routine, low inflation pressures rather than at unusually high ones. Gastric antral ultrasonography is now an established bedside tool for evaluating gastric content and volume in anesthesia [17], and gastric insufflation is itself an undesirable effect of FMV [1]. We deliberately refrain from equating this sonographic sign with clinical aspiration or regurgitation, which we did not measure; the value of avoiding gastric insufflation does not depend on that extrapolation. The pre-specified comparison of antral area across all patients did not differ between groups because insufflation was confined to this minority, leaving the group distributions largely overlapping—a pattern evident in the distribution of the antral-area change rather than in its central value (Fig. 2). Because these binary measures were defined post hoc, they should be regarded as exploratory and hypothesis-generating.

International consensus recommendations for the surgical patient advise lung-protective ventilation, including the initiation of PEEP during induction [18]. The trial was conducted before the release of these guidelines. Omitting peri-induction ventilation forgoes induction-phase recruitment and PEEP, which might promote atelectasis; applying PEEP during facemask ventilation at induction has been shown to reduce post-induction atelectasis [19]. Conversely, omitting FMV avoids gastric insufflation. Future studies are warranted to find out the impact of omitting FMV on lung atelectasis and whether this drawback overshadows the benefit of minimizing gastric insufflation.

Although FMV may cause some gastric insufflation, clinically significant regurgitation or aspiration is rare and, when it occurs, is usually without serious sequelae in fasted elective patients (aspiration in approximately 1 in 3,216 [20] to 1 in 7,103 [21] anesthetics, with no respiratory sequelae in the majority of survivors). Conversely, omitting routine FMV inherently reduces the margin of safety in an unanticipated difficult airway, potentially complicating rescue ventilation, shortening the time available for intervention, and, as noted above, forgoing peri-induction lung protection [19]. The no-FMV strategy also requires a higher intubating dose of rocuronium (1.2 mg/kg) and, for timely reversal, sugammadex; the routine use of a larger neuromuscular-blocker dose and of sugammadex carries resource and cost implications that should be weighed against the modest reduction in gastric insufflation.

Despite the adequate preoxygenation and the availability of sugammadex for rapid reversal of neuromuscular blockade, a successful rescue in a cannot-intubate, cannot-ventilate emergency is not fully guaranteed, particularly in patients whose oxygen reserves are small [22]. This is precisely why the strategy must be confined to strictly selected low-risk patients.

### Limitation

This trial has several limitations. First, the trial stopped at the interim analysis with zero events, so it did not reach the sample required to establish non-inferiority formally; therefore, we do not rest the paper’s conclusions on the formal non-inferiority result. Second, the participants were low-risk, predominantly female, and drawn from a single center undergoing elective thyroid surgery; the predominance of women reflects this surgical population as well as the low-risk patients for difficult airway. Third, the gastric insufflation measures were defined post hoc and represent a surrogate sonographic sign rather than a clinical outcome. Fourth, we did not measure atelectasis. Fifth, the operator-reported ratings were exploratory, unvalidated, and unblinded. Sixth, hemodynamic and arterial blood-gas data (PaO₂, PaCO₂) were not collected; these would be a valuable addition to future confirmatory trials

Adequately powered trials in broader and higher-risk populations, and trials incorporating apneic oxygenation [23] and measures of atelectasis and clinical aspiration, are needed to define how widely FMV can be safely omitted.

## Conclusions

In low-risk adults undergoing elective thyroid surgery, no episode of oxygen desaturation occurred whether or not FMV was performed before tracheal intubation. Because the trial was stopped at the interim analysis with zero events in both groups, formal statistical non-inferiority was not demonstrated. These findings are therefore best interpreted as clinically reassuring but not confirmatory. Gastric insufflation was confined to patients who received FMV. Together, these results invite a reconsideration of routine FMV in strictly selected low-risk patients but call for confirmation in larger and more diverse populations.

## Data Availability

The datasets used and/or analysed during the current study are available from the corresponding author on reasonable request. The full trial protocol, the August 2015 protocol amendment that added the interim analysis, and the statistical analysis plan are likewise available from the corresponding author on reasonable request.

## Abbreviations

AP: Anteroposterior
ASA: American Society of Anesthesiologists
CC: Craniocaudal
CI: Confidence interval
CSA: Cross-sectional area
FMV: Face-mask ventilation
IQR: Interquartile range
PEEP: Positive end-expiratory pressure
RD: Risk difference
SpO₂: Peripheral oxygen saturation
TOF: Train-of-four
VAS: Visual analog scale
